# Supplementary material for: Mutations in PCYT2 disrupt etherlipid biosynthesis and cause a complex hereditary spastic paraplegia
Source: Brain. 2019 Oct 22;142(11):3382–97. doi: 10.1093/brain/awz291 (PMC6821184; doi:10.1093/brain/awz291)
Supplement: awz291_Supplementary_Data [file awz291_supplementary_data.zip › awz291-Suppl_data/Supplementary_Data4.zip!/Supplementary information 4 - plasma lipidomics.zip!/Plasma lipidomics/Control vs Patient2/Statistical plots and data/Heatmap sorted on p-value and FC.pdf]

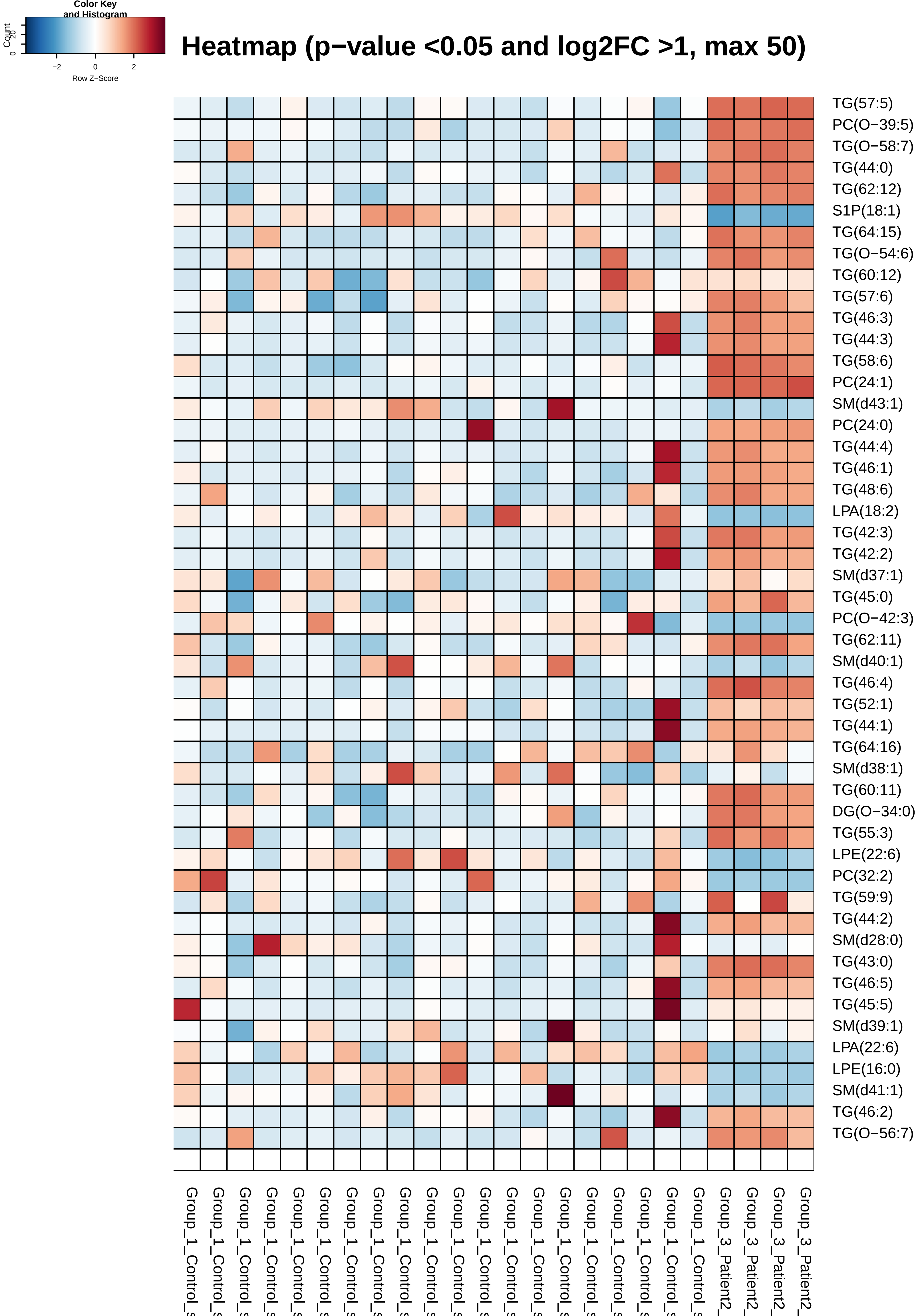

Color Key
and Histogram
Count
0
20
−2
0
2
Row Z−Score
Heatmap (p−value <0.05 and log2FC >1, max 50)
TG(57:5)
PC(O−39:5)
TG(O−58:7)
TG(44:0)
TG(62:12)
S1P(18:1)
TG(64:15)
TG(O−54:6)
TG(60:12)
TG(57:6)
TG(46:3)
TG(44:3)
TG(58:6)
PC(24:1)
SM(d43:1)
PC(24:0)
TG(44:4)
TG(46:1)
TG(48:6)
LPA(18:2)
TG(42:3)
TG(42:2)
SM(d37:1)
TG(45:0)
PC(O−42:3)
TG(62:11)
SM(d40:1)
TG(46:4)
TG(52:1)
TG(44:1)
TG(64:16)
SM(d38:1)
TG(60:11)
DG(O−34:0)
TG(55:3)
LPE(22:6)
PC(32:2)
TG(59:9)
TG(44:2)
SM(d28:0)
TG(43:0)
TG(46:5)
TG(45:5)
SM(d39:1)
LPA(22:6)
LPE(16:0)
SM(d41:1)
TG(46:2)
TG(O−56:7)
